# Supplementary material for: Inflammatory Biomarker Score Identifies Patients with Six-Fold Increased Risk of One-Year Mortality after Pancreatic Cancer
Source: Cancers (Basel). 2021 Sep 13;13(18):4599. doi: 10.3390/cancers13184599 (PMC8466571; doi:10.3390/cancers13184599)
Supplement: Supplementary file 1 [file cancers-13-04599-s001.zip › cancers-1359225-supplementary.pdf]

SUPPLEMENTARY MATERIAL for

Inflammatory biomarker score identifies patients with six-fold increased risk of one-year mortality after pancreatic cancer

Kjaergaard AD, Chen IM, Johansen AZ, Nordestgaard BG, Bojesen SE, Johansen JS.

TABLE OF CONTENTS:

Supplementary Introduction

Supplementary Methods

Supplementary References

Table S1. Characteristics of patients with pancreatic ductal adenocarcinoma according to plasma YKL-40 categories.

Table S2. Characteristics of patients with pancreatic ductal adenocarcinoma according to *CHI3L1* rs4950928.

Table S3. Risk of one-year mortality after pancreatic ductal adenocarcinoma according to pairwise combinations of low, intermediate and high CRP, CA19-9, IL-6 and YKL-40 levels stratified by operation status.

Table S4. Risk of one-year mortality after pancreatic ductal adenocarcinoma according to a combination of low and high CRP and CA19-9 levels stratified by operation status.

Figure S1. Kaplan-Meier survivor function stratified by performance status (PS), operation and pancreatic ductal adenocarcinoma stage for plasma CRP categories.

Figure S2. Kaplan-Meier survivor function stratified by performance status (PS), operation and pancreatic ductal adenocarcinoma stage for plasma CA-19-9 categories.

Figure S3. Kaplan-Meier survivor function stratified by performance status (PS), operation and pancreatic ductal adenocarcinoma stage for plasma IL-6 categories.

Figure S4. Kaplan-Meier survivor function stratified by performance status (PS), operation and pancreatic ductal adenocarcinoma stage for plasma YKL-40 categories.

Figure S5. Risk of one-year mortality after diagnosis of pancreatic ductal adenocarcinoma per doubling in biomarker levels.

Figure S6. Kaplan-Meier survival curves for *CHI3L1* rs4950928 genotype stratified by performance status (PS), operation and pancreatic ductal adenocarcinoma stage.

Figure S7 Area under receiver operating curves (AUROC) for one-year mortality for CRP, CA 19-9, IL-6 and YKL-40 stratified by performance status (PS), operation and pancreatic ductal adenocarcinoma (PDAC) stage.

Figure S8. Risk of one-year mortality after diagnosis of pancreatic ductal adenocarcinoma according to biomarker score (the sum of CRP, CA 19-9, IL-6 and YKL-40 coded as 0, 1 and 2 for low, intermediate and high plasma levels respectively.

Figure S9. Risk of one-year mortality after diagnosis of pancreatic ductal adenocarcinoma according to biomarker score (the sum of CRP, CA 19-9, and IL-6 coded as 0, 1 and 2 for low, intermediate and high plasma levels respectively and operation.

Figure S10. Median plasma YKL-40 by *CHI3L1* rs4950928 genotype and population.

Figure S11. Median levels of C-reactive protein, interleukin-6 and CA 19-9 across the plasma YKL-40 categories.

Supplementary Introduction

YKL-40, also named chitinase-3-like-1 protein, is a glycoprotein that plays a role in inflammation, remodeling of the extracellular matrix, angiogenesis, metastasis and protection against apoptosis (1-6). YKL-40 is produced by cancer-, immune- and stromal cells. Expression of YKL-40 is induced by cytokines such as IL-13, IL-6 and IL-1β, whereas miR-24, miR-449a and miR-342-3p can inhibit the expression (7). YKL-40 serves as a binding partner for carbohydrate residues of the glycoconjugates of the extracellular matrix and the cell, and it can also interact with receptors IL-13Rα2, CRTH, PAR-2, CD44, syndecan-1 and RAGE triggering signaling cascades related to ERK, MAPK and AKT (8-10).

We tested the hypothesis that measured and genetically predicted elevated plasma YKL-40 levels are associated with poor survival in patients with pancreatic ductal adenocarcinoma (PDAC). For genetically predicted elevated plasma YKL-40 levels, we employed the genotype for rs4950928, a single nucleotide polymorphism (SNP) in *CHI3L1*, chitinase-3-like-1, the gene encoding YKL-40. *CHI3L1* rs4950928 is a promoter SNP that is a genetic marker of lifelong elevated plasma YKL-40 levels (two-fold for CG and three-fold for CC versus GG genotype) (11-12). This is relevant because a Taiwanese case-study of 343 patients with hepatocellular carcinoma (HCC) and 686 cancer-free controls suggested that *CHI3L1* rs4950928 may be a prognostic biomarker for HCC because it was associated with vascular invasion (13). Furthermore, *CHI3L2* rs684559, a SNP in a closely related human member of chitinase-like family was recently identified as a possible prognostic marker for survival in 331 patients after PDAC resection (14).

Supplementary Methods

Genotyping of CHI3L1 rs4950928A

Genotyping of *CHI3L1* rs4950928 was performed in 992 of the patients using Taq-Man (Applied Biosystems by Life Technologies Corporation, Carlsbad, CA) assays. We used a forward (AGT TCC CAT AAA AGG GCT GGT TT) and a reverse (CCC AGG CCC TGT ACT TCC TTT ATA T) primer for the PCR amplification of leukocyte DNA and common (CTCCCCCACGCGGC) and variant (ACTCCCCGACGCGGC) probes to determine genotype.

We examined the association between rs4950928 genotype and plasma YKL-40 in patients with PDAC and compared this with the genotype-phenotype association in the Danish general population of 21,166 individuals (11-12). Interaction was examined by likelihood ratio test after linear regression between the models with and without an interaction term. We investigated potential interaction between genotype and population (PDAC or general population) with regards to plasma YKL-40 levels, as well as potential interactions between biomarkers and PS (PS=0 versus PS≥1), operation (yes/no) and PDAC stage (stages I-II versus III-IV) with regards to survival.

The differences across *CHI3L1* rs4950928 genotype and other categories of covariates were examined using log-rank trend tests. We used Cox regression analysis (with time since blood sampling as the time scale) to calculate hazard ratios and 95% confidence intervals (CIs) for one-year mortality after PDAC.

Supplementary Results

Increasing plasma YKL-40 levels were associated with increasing levels of CRP, CA 19-9 and IL-6 (Supplementary Table S1). *CHI3L1* rs4950928 genotype was not associated with any of the baseline characteristics except plasma YKL-40 levels (Supplementary Table S2).

Median plasma YKL-40 increased with each additional C-allele (Supplementary Figure S10). Compared to the GG genotype, median plasma YKL-40 levels in patients with PDAC were 143% and 275% higher in the CG and CC genotypes. In comparison, corresponding estimates in the Danish general population were lower: 95% for the CG and 195% for the CC genotype (Supplementary Figure S10, p-value for interaction=$5x10^{-28}$).

In corresponding analyses by rs4950928 genotype, we did not observe associations with the cumulative survival in any of the strata or overall (Supplementary Figure S6). In overall tests for trend of survivor function p-values were 0.13 for one-year survival and 0.18 for five-year survival.

Increasing plasma YKL-40 categories were associated with increased median levels of CRP, CA 19-9 and IL-6 (Supplementary Figure S11). Compared to low plasma YKL-40 category (<200 µg/L), intermediate YKL-40 category (200-799 µg/L) was associated with an increase in median levels of CRP by 313%, CA-19-9 by 86% and IL-6 by 134%. Correspondingly, high YKL-40 category (>800 µg/L) was associated with an increase in median levels of CRP by 1033%, CA 19-9 by 70% and IL-6 by 485% (Supplementary Figure S11).

Supplementary References

1. Libreros S, Iragavarapu-Charyulu V. YKL-40/CHI3L1 drives inflammation on the road of tumor progression. J Leukoc Biol. 2015;98:931-6.
2. Shao R, Taylor SL, Oh DS, Schwartz LM. Vascular heterogeneity and targeting: the role of YKL-40 in glioblastoma vascularization. Oncotarget. 2015;6:40507-18.
3. Lee CG, Da Silva CA, Dela Cruz CS, Ahangari F, Ma B, Kang MJ, et al. Role of chitin and chitinase/chitinase-like proteins in inflammation, tissue remodeling, and injury. Annu Rev Physiol. 2011;73:479-501.
4. Chen Y, Zhang S, Wang Q, Zhang X. Tumor-recruited M2 macrophages promote gastric and breast cancer metastasis via M2 macrophage-secreted CHI3L1 protein. J Hematol Oncol. 2017;10:36.
5. Kim DH, Park HJ, Lim S, Koo JH, Lee HG, Choi JO, et al. Regulation of chitinase-3-like-1 in T cell elicits Th1 and cytotoxic responses to inhibit lung metastasis. Nat Commun. 2018;9:503.
6. Cohen N, Shani O, Raz Y, Sharon Y, Hoffman D, Abramovitz L, et al. Fibroblasts drive an immunosuppressive and growth-promoting microenvironment in breast cancer via secretion of Chitinase 3-like 1. Oncogene. 2017;36:4457-68.
7. Yeo IJ, Lee CK, Han SB, Yun J, Hong JT. Roles of chitinase 3-like 1 in the development of cancer, neurodegenerative diseases, and inflammatory diseases. Pharmacol Ther. 2019;203:107394.
8. Zhao T, Su Z, Li Y, Zhang X, You Q. Chitinase-3 like-protein-1 function and its role in diseases. Signal Transduct Target Ther. 2020;5:201.

9. Ngernyuang N, Yan W, Schwartz LM, Oh D, Liu YB, Chen H, et al. A Heparin Binding Motif Rich in Arginine and Lysine is the Functional Domain of YKL-40. Neoplasia. 2018;20:182-92.
10. Geng B, Pan J, Zhao T, Ji J, Zhang C, Che Y, et al. Chitinase 3-like 1-CD44 interaction promotes metastasis and epithelial-to-mesenchymal transition through beta-catenin/Erk/Akt signaling in gastric cancer. J Exp Clin Cancer Res. 2018;37:208.
11. Kjaergaard AD, Bojesen SE, Nordestgaard BG, Johansen JS. YKL-40 and alcoholic liver and pancreas damage and disease in 86,258 individuals from the general population: cohort and mendelian randomization studies. Clin Chem. 2014;60:1429-40.
12. Kjaergaard AD, Nordestgaard BG, Johansen JS, Bojesen SE. Observational and genetic plasma YKL-40 and cancer in 96,099 individuals from the general population. Int J Cancer. 2015;137:2696-704.
13. Huang WS, Lin HY, Yeh CB, Chen LY, Chou YE, Yang SF, et al. Correlation of Chitinase 3-Like 1 Single Nucleotide Polymorphisms with Hepatocellular Carcinoma in Taiwan. Int J Med Sci. 2017;14:136-42.
14. Dimitrakopoulos C, Vrugt B, Flury R, Schraml P, Knippschild U, Wild P, et al. Identification and Validation of a Biomarker Signature in Patients With Resectable Pancreatic Cancer via Genome-Wide Screening for Functional Genetic Variants. JAMA Surg. 2019;154:e190484.

Table S1. Characteristics of patients with pancreatic ductal adenocarcinoma according to plasma YKL-40 categories.

		Plasma YKL-40, µg/L			
	Missing, %	<200	200-799	≥800	P-value
Number of participants, N (%)	NA	676 (68%)	267 (27%)	50 (5%)	NA
Age, years	0	66 (61-72)	69 (64-75)	68 (60-73)	$6x10^{-4}$
Women, %	0	48	40	34	0.02
Eversmoker, %	10	60	62	70	0.70
Body mass index, kg/m^2	5.8	22.9 (20.7-25.8)	23.2 (20.9-26.0)	22.9 (20.6-25.4)	0.69
High alcohol consumption, %	10	21	26	30	0.35
Performance status	9.7	1 (0-1)	1 (0-1)	1 (1-2)	$1x10^{-4}$
Charlson comorbidity index	3.7	3 (2-4)	3 (2-4)	3 (2-4)	$4x10^{-5}$
Diabetes, %	1.8	24	25	48	0.004
Operated, %	0	33	23	24	0.006
Metastasing cancer, %	0.5	43	63	74	$5x10^{-9}$

Tumour size, mm	13	3.5 (2.5-4.5)	3.5 (2.5-5.0)	4.0 (3.0-5.5)	0.23
Stage I-II, %	1.5	22	19	18	
Stage III, %	0.8	33	17	8	$7x10^{-9}$
Stage IV, %	0	44	64	74	
C-reactive protein, mg/L	1.8	5.6 (3.0-20)	23 (5.2-668)	63 (24-112)	$61x10^{-28}$
CA 19-9, kU/L	1.6	393 (55-2260)	732 (73-5524)	668 (62-18000)	0.003
Interleukin-6, µg/L	0	4.1 (2.3-7.5)	9.6 (4.9-21)	24 (8.6-38)	$13x10^{-38}$

Values collected at inclusion during July 3rd 2008 through August 24th 2017 are expressed as numbers of participants, frequencies, or medians (interquartile ranges). Numbers of participants across listed variables vary slightly because we did not have information on all participants for all listed variables.

P-value is from Pearson's χ^2 or Cuzick's nonparametric test for trend. NA=not applicable.

Table S2. Characteristics of patients with pancreatic ductal adenocarcinoma according to *CHI3L1* rs4950928

	CHI3L1 rs4950928 genotype				
	Missing, %	GG	CG	CC	P-value
Number of participants, N (%)	NA	38 (4)	346 (35)	608 (61)	0.59*
Age, years	0	67 (62-73)	66 (61-72)	68 (61-73)	0.65
Women, %	0	68	53	54	0.20
Eversmoker, %	10	74	62	60	0.21
Body mass index, kg/m^2	5.8	23.4 (22.0-25.6)	22.4 (20.2-25.7)	23.3 (21.0-25.8)	0.19
High alcohol consumption, %	10	13	22	24	0.23
Performance status	9.7	1 (0-1)	1 (0-1)	1 (0-1)	0.43
Charlson comorbidity index	3.8	3 (2-4)	3 (2-4)	3 (2-4)	0.24
Diabetes, %	1.8	34	24	26	0.52
Operated, %	0	26	29	31	0.66
Metastasing cancer, %	0.5	50	51	50	0.97

Tumour size, mm	13	3.5 (2.7-5.0)	3.5 (2.7-4.8)	3.5 (2.5-4.7)	0.73
Stage I-II, %	0	13	19	23	
Stage III, %	1.7	37	27	27	0.44
Stage IV, %	0.8	50	52	50	
C-reactive protein, mg/L	2.1	9.5 (3.0-39)	10 (3.0-41)	8.1 (3.0-34)	0.20
CA 19-9, kU/L	1.7	1747 (221-7560)	580 (70-3300)	373 (54-2999)	0.03
Interleukin-6, µg/L	0.2	6.4 (2.9-14)	5.6 (2.7-13)	5.1 (2.6-11)	0.22
YKL-40, ng/L	0.3	45 (29-75)	108 (63-174)	167 (101-266)	$5x10^{-29}$

Values collected at inclusion during July 3rd 2008 through August 24th 2017 are expressed as numbers of participants, frequencies, or medians (interquartile ranges).

P-value is from Pearson's χ^2 (*Hardy-Weinberg equilibrium) or Cuzick's nonparametric test for trend. NA=not applicable.

Table S3. Risk of one-year mortality after pancreatic ductal adenocarcinoma according to pairwise combinations of low, intermediate and high CRP, CA19-9, IL-6 and YKL-40 levels stratified by operation status.

	Hazard ratios (95% confidence intervals)		
Biomarker score	All	Operated	Not operated
CRP and CA19-9			
score=0	reference	reference	reference
score=1	1.76 (1.20-2.59)	4.12 (1.76-9.68)	1.27 (0.83-1.95)
score=2	3.02 (2.05-4.44)	6.83 (2.80-16.64)	2.21 (1.45-3.37)
score=3	5.02 (3.34-7.54)	10.48 (3.36-32.62)	3.60 (2.31-5.59)
score=4	4.64 (2.60-8.30)	36.82 (3.55-381.83)	3.35 (1.82-6.15)
CRP and IL-6			
score=0	reference	reference	reference
score=1	1.48 (1.16-1.88)	1.65 (0.99-2.75)	1.43 (1.08-1.88)
score=2	2.44 (1.96-3.03)	2.59 (1.49-4.51)	2.35 (1.85-2.98)
score=3	3.19 (2.37-4.30)	6.16 (2.84-13.39)	2.90 (2.09-4.01)
score=4	4.69 (3.29-6.68)	32.68 (3.24-329.50)	4.18 (2.87-6. 09)
CRP and YKL-40			
score=0	reference	reference	reference

score=1	1.50 (1.22-1.83)	1.69 (1.06-2.69)	1.44 (1.15-1.81)
score=2	2.08 (1.65-2.62)	1.50 (0.76-2.97)	2.11 (1.64-2.71)
score=3	2.87 (2.14-3.87)	2.48 (0.89-6.95)	2.75 (1.99-3.79)
score=4	2.86 (1.56-5.24)	NC	3.04 (1.64-5.62)
CA19-9 and IL-6			
score=0	reference	reference	reference
score=1	1.60 (1.08-2.36)	3.49 (1.48-8.27)	1.12 (0.72-1.74)
score=2	2.85 (1.93-4.20)	7.91 (3.30-18.95)	1.95 (1.27-3.00)
score=3	4.60 (3.04-6.97)	17.68 (5.84-53.56)	3.03 (1.93-4.74)
score=4	6.43 (3.69-11.20)	NC	4.45 (2.50-7.94)
CA19-9 and YKL-40			
score=0	reference	reference	reference
score=1	1.54 (1.12-2.11)	3.96 (1.90-8.29)	1.13 (0.80-1.60)
score=2	2.10 (1.50-2.93)	3.49 (1.48-8.25)	1.65 (1.15-2.37)
score=3	3.28 (2.21-4.86)	5.42(1.74-16.91)	2.59 (1.70-3.94)
score=4	2.64 (1.40-4.99)	NC	2.10 (1.10-4.01)
IL-6 and YKL-40			
score=0	reference	reference	reference

score=1	1.62 (1.32-1.99)	2.74 (1.69-4.45)	1.51 (1.20-1.89)
score=2	2.16 (1.72-2.72)	2.03 (1.10-3.77)	2.14 (1.67-2.75)
score=3	2.79 (2.03-3.83)	0.81 (0.18-3.54)	2.90 (2.06-4.09)
score=4	3.03 (1.89-4.87)	7.92 (2.38-26.38)	2.68 (1.60-4.49)

We investigated pairwise combinations of CRP, CA19-9, IL-6 and YKL-40, as the sum of two biomarkers at a time. As the biomarkers were coded 0, 1 and 2 for low, intermediate and high biomarker categories, each patient received a score between 0 (if both biomarkers were low) and 4 (if both biomarkers were high).

Hazard ratios were adjusted for age, sex, performance status (PS 0, 1 and 2-4), operation (yes/no) and cancer stage (I, II and III-IV).

NC: not computable.

Table S4. Risk of one-year mortality after pancreatic ductal adenocarcinoma according to a combination of low and high CRP and CA19-9 levels stratified by operation status.

	Hazard ratios (95% confidence intervals)		
Biomarker levels	All	Operated	Not operated
CRP<3 & CA19-9≤37	reference	reference	reference
CRP≥3 or CA19-9<37	1.50 (0.75-2.99)	1.92 (0.57-6.50)	1.18 (0.51-2.75)
CRP≥3 & CA19-9>37	2.62(1.34-5.13)	5.13 (1.58-16.65)	1.86 (0.82-4.23)

Hazard ratios were adjusted for age, sex, performance status (PS 0, 1 and 2-4), operation (yes/no) and cancer stage (I, II and III-IV).

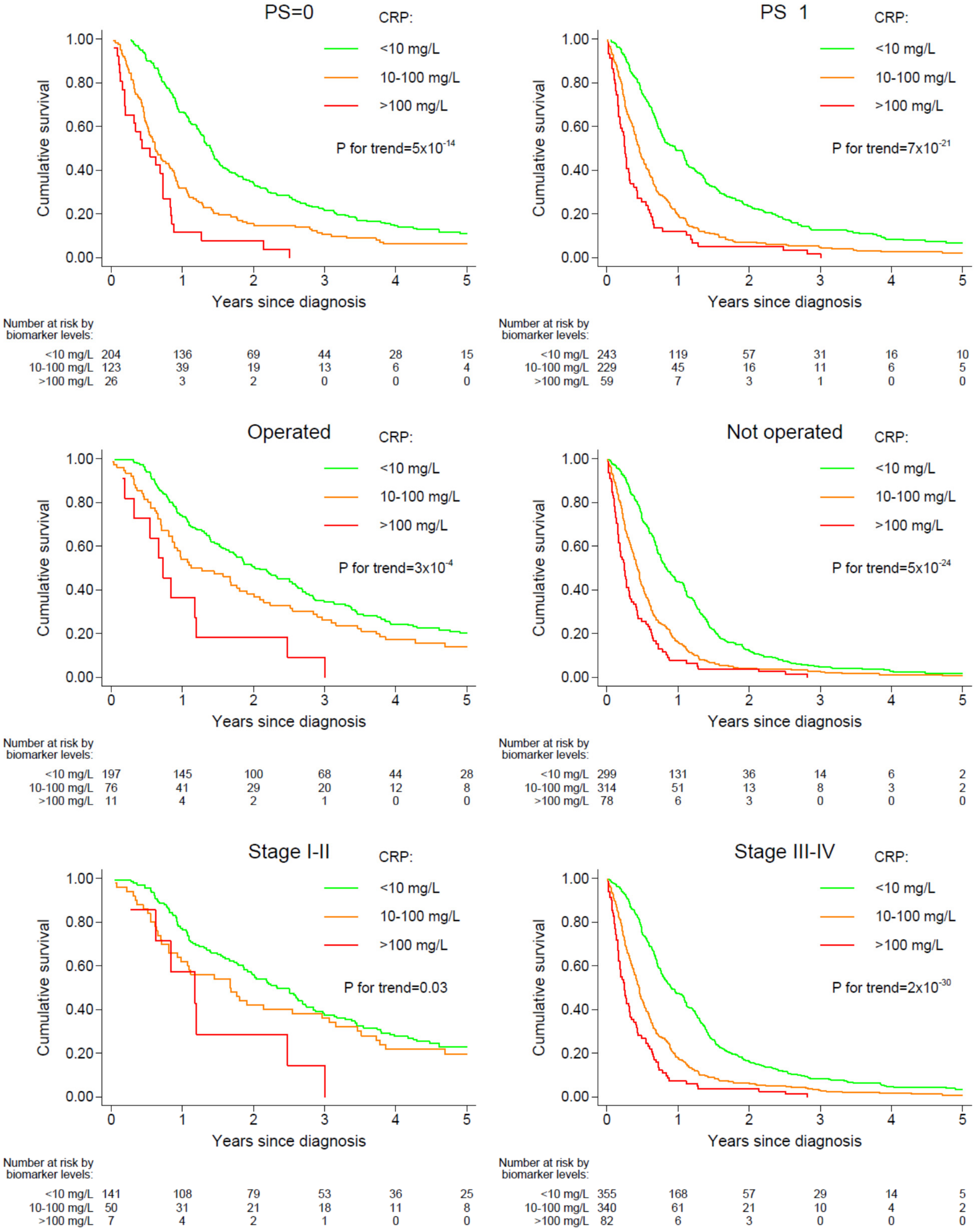

Figure S1. Kaplan-Meier survivor function stratified by performance status (PS), operation and pancreatic ductal adenocarcinoma stage for plasma CRP categories.

P for trend is from Wald test of trend across groups.

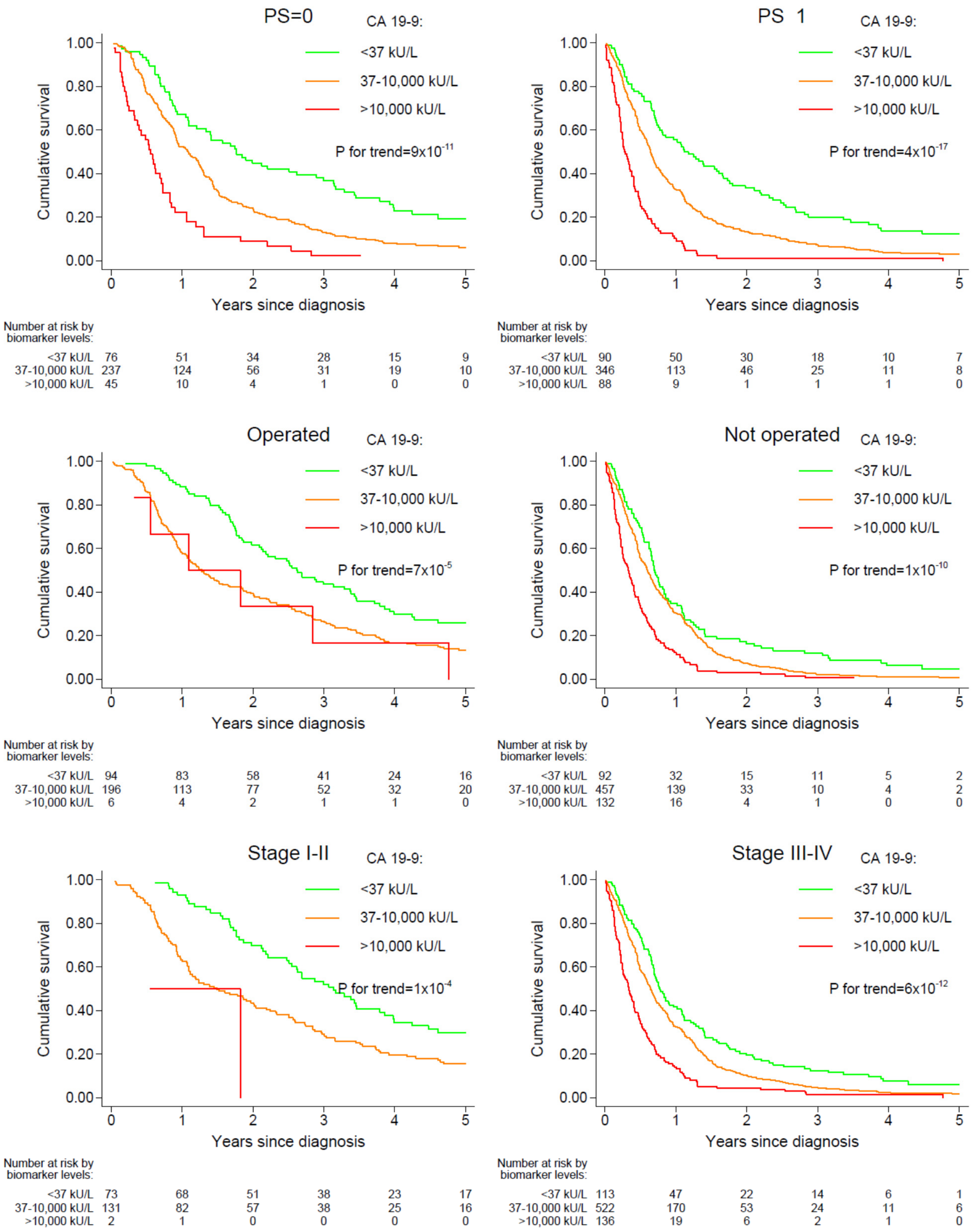

Figure S2. Kaplan-Meier survivor function stratified by performance status (PS), operation and pancreatic ductal adenocarcinoma stage for plasma CA 19-9 categories.

P for trend is from Wald test of trend across groups.

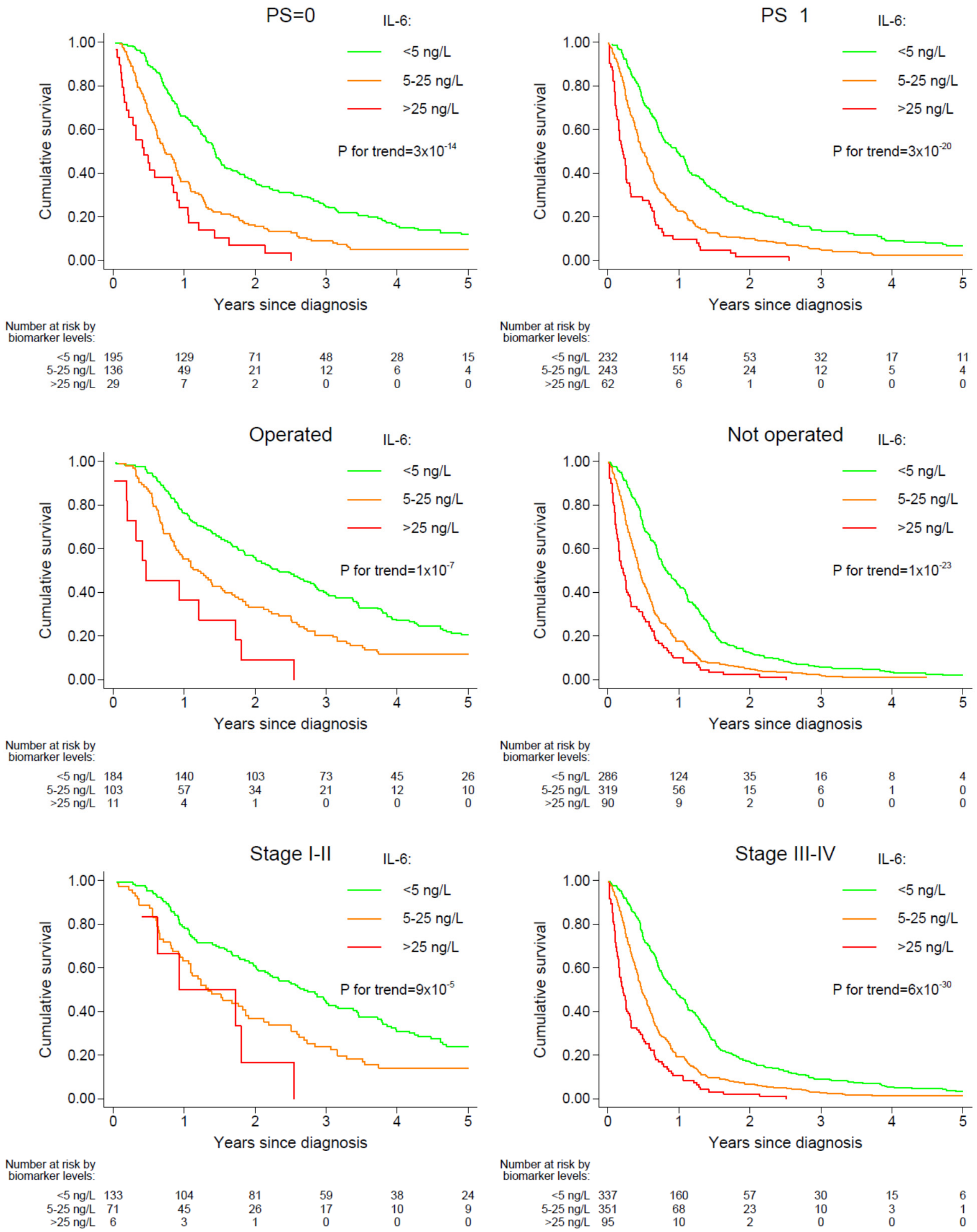

Figure S3. Kaplan-Meier survivor function stratified by performance status (PS), operation and pancreatic ductal adenocarcinoma stage for plasma IL-6 categories.

P for trend is from Wald test of trend across groups.

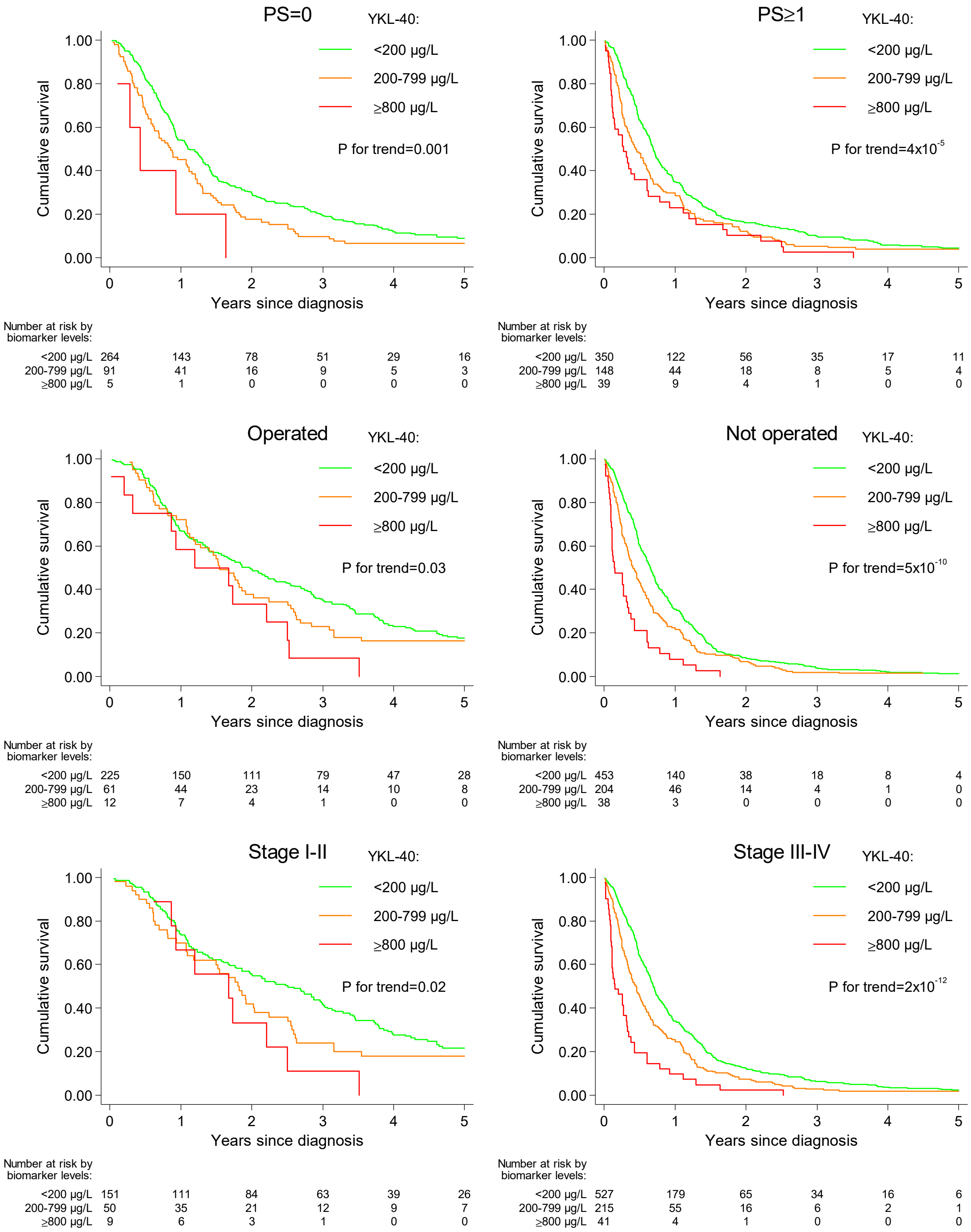

Figure S4. Kaplan-Meier survivor function stratified by performance status (PS), operation and pancreatic ductal adenocarcinoma stage for plasma YKL-40 categories.

P for trend is from Wald test of trend across groups.

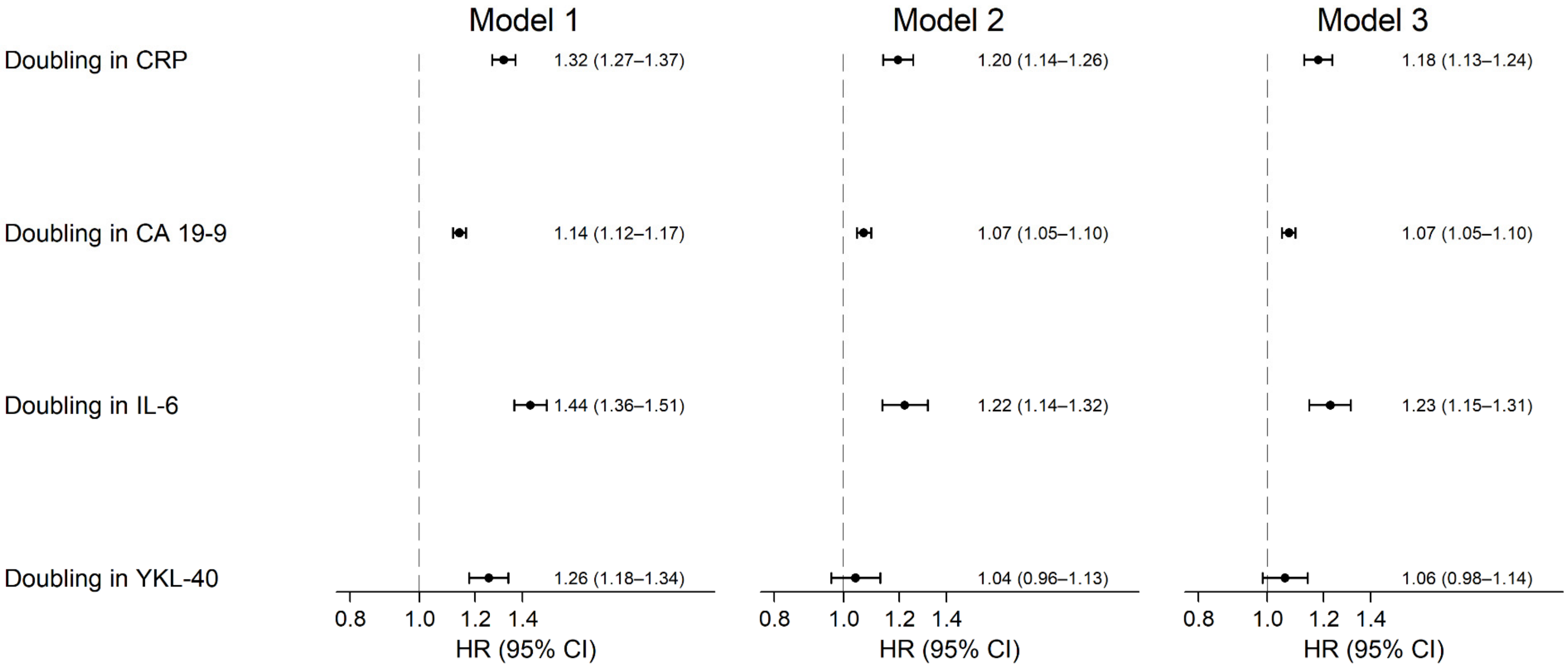

Figure S5. Risk of 1-year mortality after pancreatic ductal adenocarcinoma per doubling in biomarker levels.
Model 1 was adjusted for age and sex, and included all patients.
Models 2 and 3 were adjusted for, age, sex operation, performance status, cancer stage and all the biomarkers (except if considered exposure).
Model 2 included patients with complete information only. Model 3 included all patients, as we performed multiple imputation on the very few missing values.

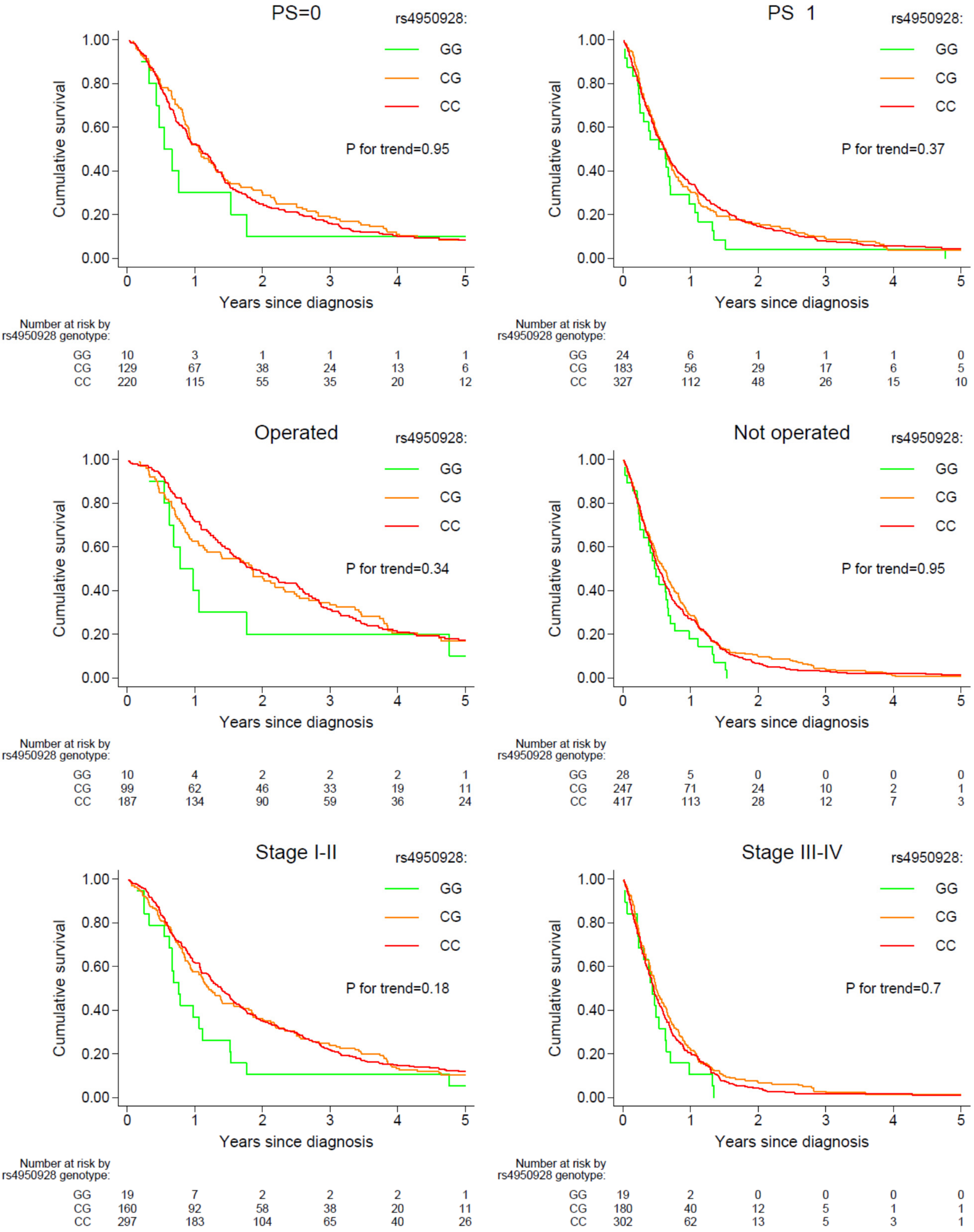

Figure S6. Kaplan-Meier survival curves for *CHI3L1* rs4950928 genotype stratified by performance status (PS), operation and pancreatic ductal adenocarcinoma stage.

P for trend is from Wald test of trend across groups.

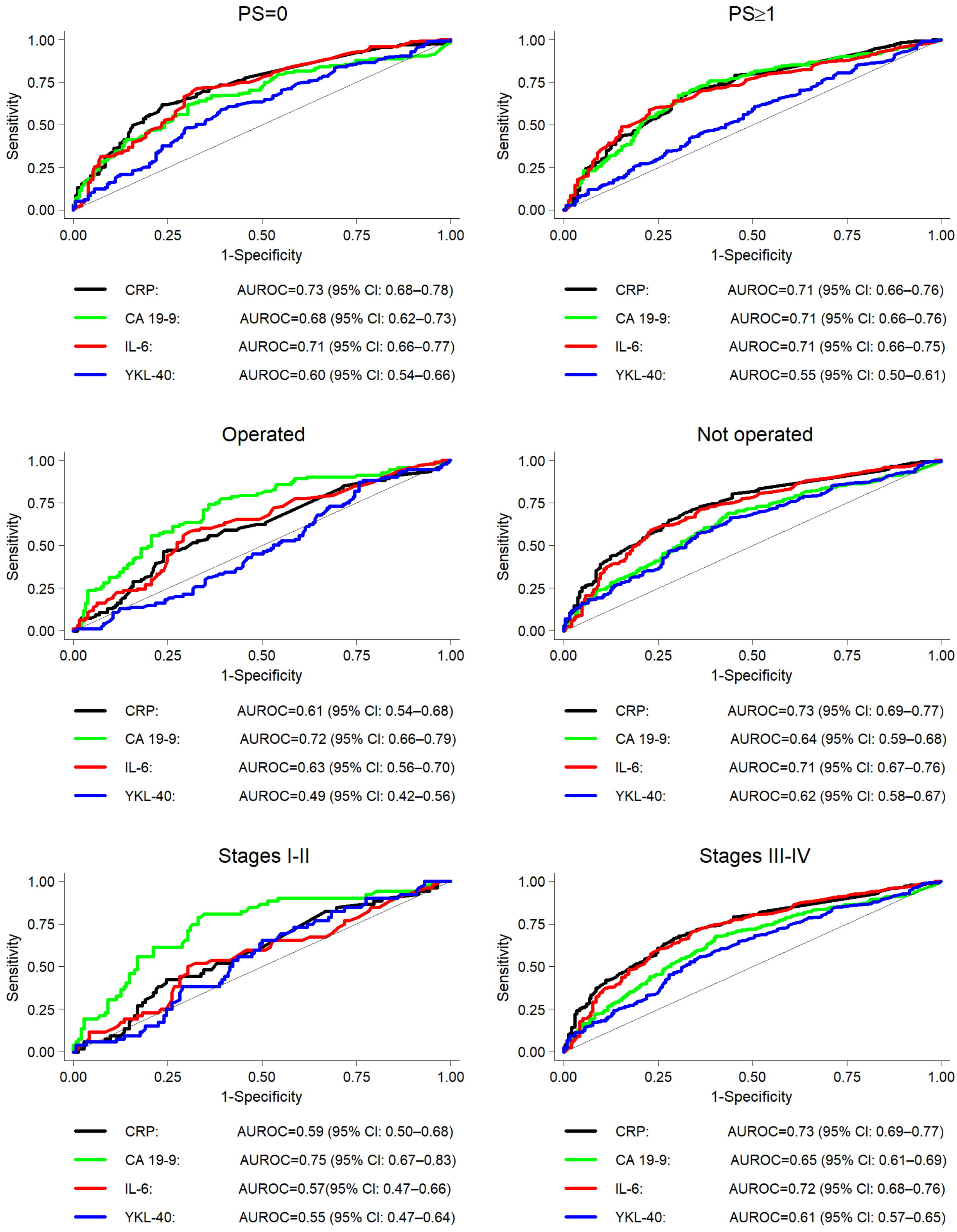

Figure S7. Area under receiver operating curves (AUROC) for one-year mortality for CRP, CA 19-9 IL-6 and YKL-40 stratified by performance status (PS), operation and pancreatic ductal adenocarcinoma (PDAC) stage. Analyses were restricted to 959 patients with measurements of all four biomarkers

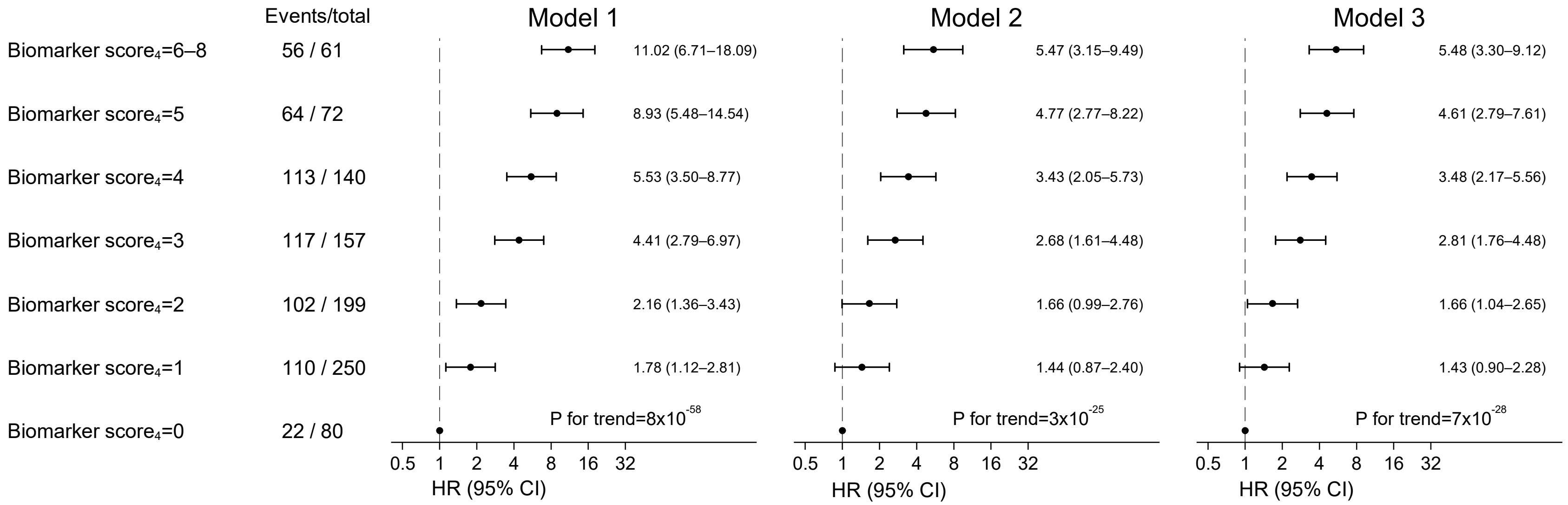

Figure S8. Risk of one-year mortality after pancreatic ductal adenocarcinoma according to biomarker score$_4$ (the sum of CRP, CA 19-9, IL-6 and YKL-40 coded as 0, 1 and 2 for low, intermediate and high plasma levels, respectively).
Model 1 was adjusted for age and sex, and included all patients.
Models 2 and 3 were adjusted for, age, sex operation, performance status and cancer stage.
Model 2 included patients with complete information only. Model 3 included all patients, as we performed multiple imputation on the very few missing values.
P for trend is from Wald test of trend across groups.

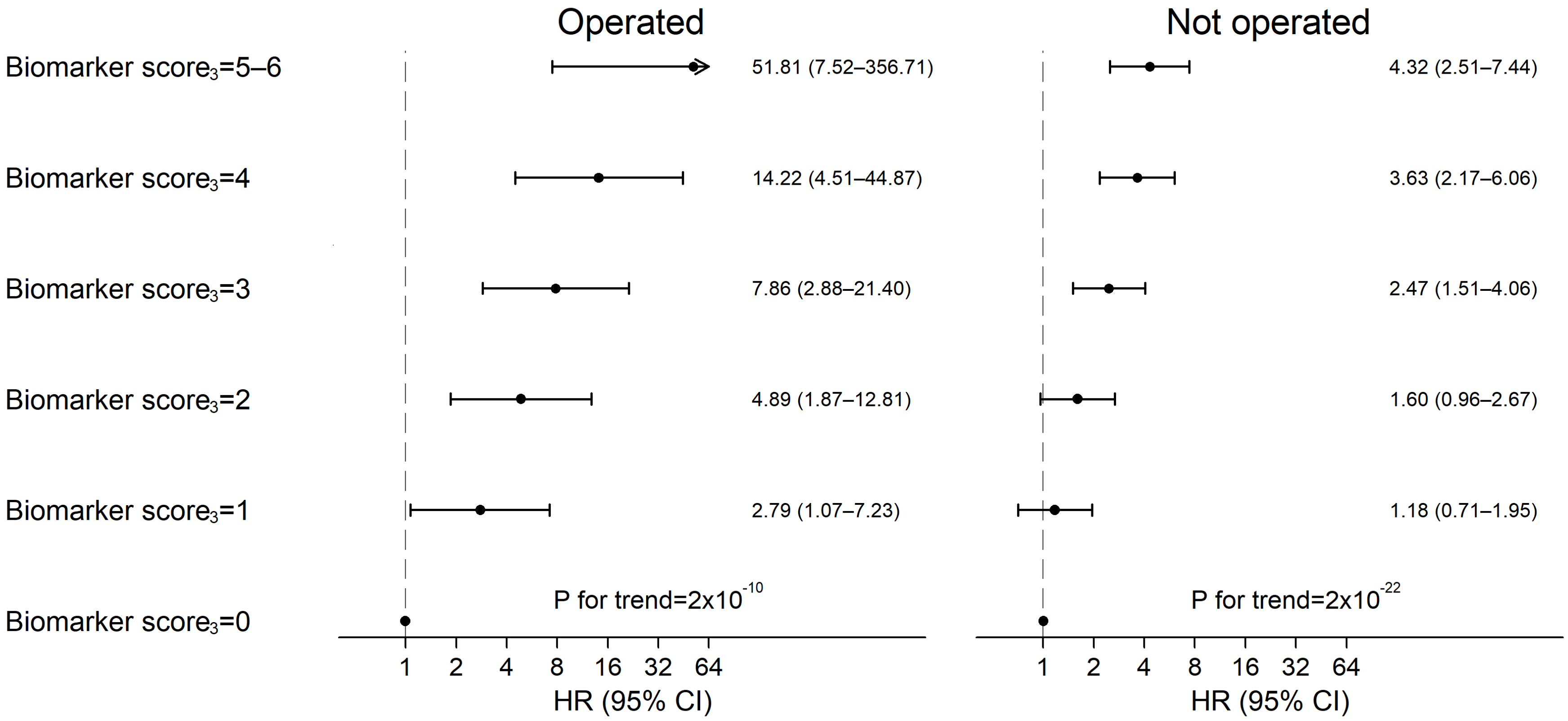

Figure S9. Risk of one-year mortality after pancreatic ductal adenocarcinoma according to biomarker score$_3$ (the sum of CRP, CA 19-9 and IL-6 coded as 0, 1 and 2 for low, intermediate and high plasma levels, respectively) and operation. Models were adjusted for age, sex, performance status and cancer stage, and included all patients, as we performed multiple imputations on very few missing values.
P for trend is from Wald test across groups treating biomarker levels as continuous variables in the Cox regression model.

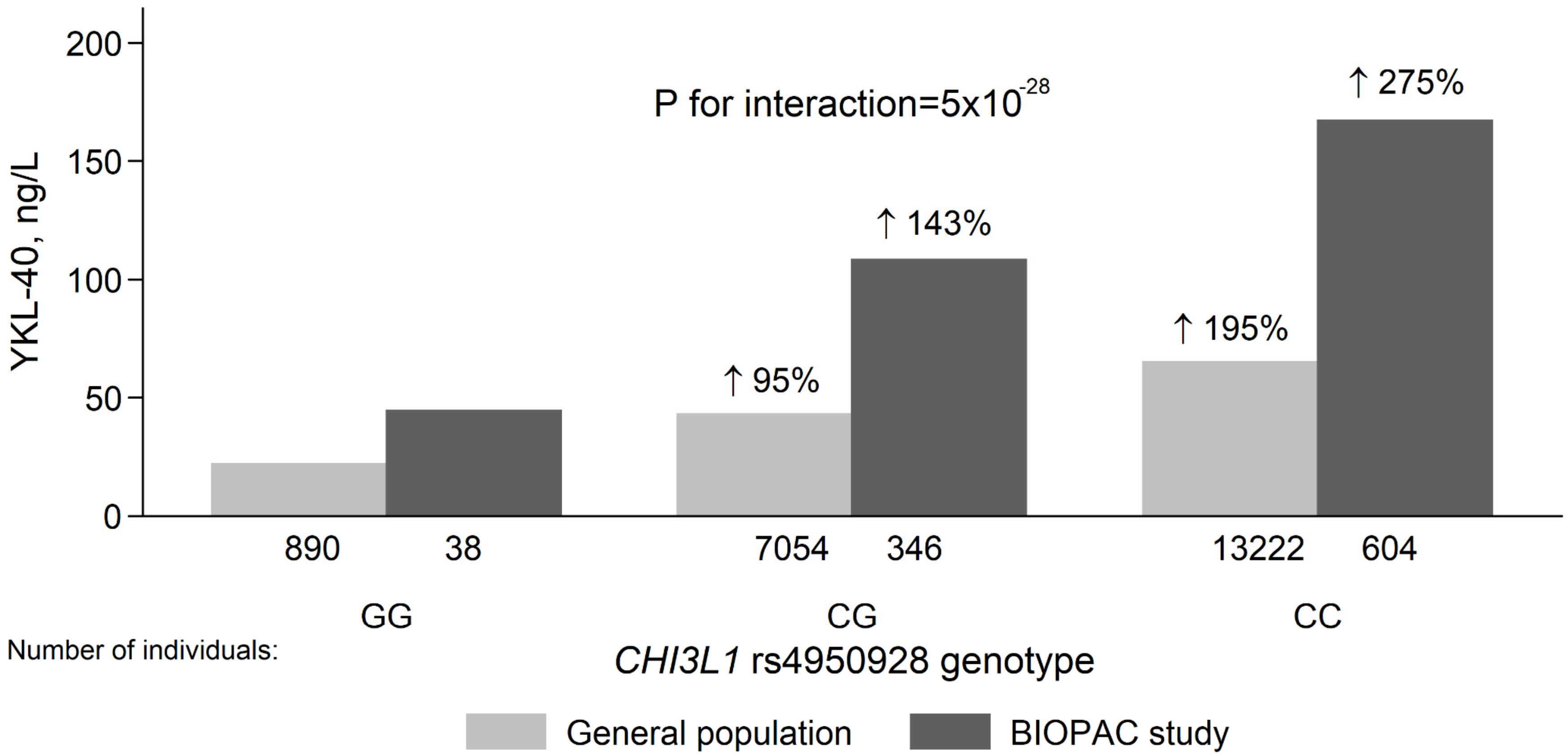

Figure S10. Median plasma YKL-40 by *CHI3L1* rs4950928 genotype and population.
Arrows represent increase in YKL-40 for intermediate (CG) and high (CC) compared to the low YKL-40 genotype (GG) for the general population (light grey) and patients with pancreatic ductal adenocarcinoma from the BIOPAC study (dark grey).

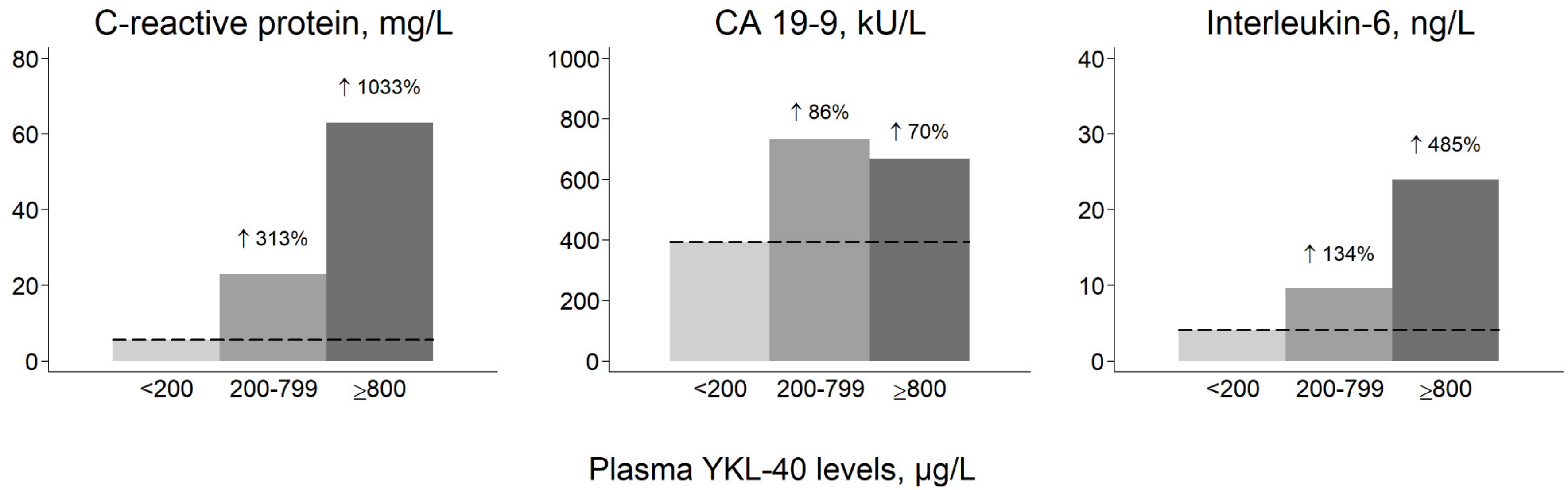

Figure S11. Median levels of C-reactive protein, Interleukin-6 and CA 19-9 across the three plasma YKL-40 categories. Arrows represent increase in biomarkers for intermediate (200-799 μg/L) and high (≥800 μg/L) YKL-40 compared to the low YKL-40 category (<200 μg/L, dashed line)
